# Identification of reference genes for gene expression studies among different developmental stages of murine hearts

**DOI:** 10.1186/s12861-021-00244-6

**Published:** 2021-09-08

**Authors:** Jie Ren, Ningning Zhang, Xiangjie Li, Xiaogang Sun, Jiangping Song

**Affiliations:** State Key Laboratory of Cardiovascular Disease, Fuwai Hospital, National Center for Cardiovascular Diseases, Chinese Academy of Medical Sciences and Peking Union Medical College, 167A Beilishi Road, Xi Cheng District, Beijing, 100037 People’s Republic of China

**Keywords:** Reference genes, Heart, Development, Real-time quantitative polymerase chain reaction (RT-qPCR), Stability

## Abstract

**Background:**

Real-time quantitative polymerase chain reaction (RT-qPCR) is a widely-used standard assay for assessing gene expression. RT-qPCR data requires reference genes for normalization to make the results comparable. Therefore, the selected reference gene should be highly stable in its expression throughout the experimental datasets. So far, reports about the optimal set of reference genes in murine left ventricle (LV) across embryonic and postnatal stages are few. The objective of our research was to identify the appropriate reference genes in murine LV among different developmental stages.

**Methods:**

We investigated the gene expression profiles of 21 widely used housekeeping genes in murine LV from 7 different developmental stages (almost throughout the whole period of the mouse lifespan). The stabilities of the potential reference genes were evaluated by five methods: GeNorm, NormFinder, BestKeeper, Delta-Ct and RefFinder.

**Results:**

We proposed a set of reliable reference genes for normalization of RT-qPCR experimental data in different conditions. Furthermore, our results showed that 6 genes (*18S, Hmbs, Ubc, Psmb4, Tfrc* and *Actb*) are not recommended to be used as reference genes in murine LV development studies. The data also suggested that the *Rplp0* gene might serve as an optimal reference gene in gene expression analysis.

**Conclusions:**

Our study investigated the expression stability of the commonly used reference genes in process of LV development and maturation. We proposed a set of optimal reference genes that are suitable for accurate normalization of RT-qPCR data in specific conditions. Our findings may be helpful in future studies for investigating the gene expression patterns and mechanism of mammalian heart development.

**Supplementary Information:**

The online version contains supplementary material available at 10.1186/s12861-021-00244-6.

## Background

The heart is the first functional organ to develop in the embryo. The gene expression constantly changes during the cardiac development and maturation [1–3]. It is a strictly-regulated process, which requires precise control of gene expression. Any disruption in cardiac development may lead to congenital heart defects, which cause significant public health burdens [4]. The main function of the heart—pumping blood to make a constant supply of oxygen and nutrients for body, is mainly determined by the left ventricle (LV) condition [5, 6]. And the LV remodeling is the main manifestation of many cardiac disorders [7, 8]. Therefore, it is crucial to give special attention to the gene expression of LV during cardiac development and maturation.

Transcriptome analysis provides broad insights into the molecular regulatory networks [9, 10]. Up to now, real-time quantitative polymerase chain reaction (RT-qPCR) is still the standard assay used for quantification of gene expression with high sensitivity and accuracy [11]. RT-qPCR data needs reference genes for normalization to make the results comparable [12]. The unstably-expressed reference genes could lead to erroneous results. Thus, selection of appropriate reference gene is important in the design of a RT-qPCR experiment [13]. The reference genes should hold a high expression stability throughout all experimental datasets [14, 15]. So far, there is no single ideal reference gene appropriate for all conditions [16]. Thus, it is critical to identify suitable reference genes with relatively stable expression in the specific context.

Mice have been frequently used as mammalian models to study the cardiac development/maturation because of their physiological, genetic and anatomical similarities to humans [17]. The common reference genes for heart samples include glyceraldehyde-3-phosphate dehydrogenase (*Gapdh*), 18S ribosomal RNA (*18S*), and actin beta (*Actb*) [18]. However, in the process of heart development or maturation, the expression levels for some housekeeping genes are significantly altered [19]. A recent study has identified some appropriate reference genes for heart tissue of mice at different developmental stages. However, only a limited number of candidate reference genes and a small number of samples at different developmental stages were included [20]. So far, information about the optimal reference genes sets in murine LV tissues across embryonic and postnatal stages is still inadequate.

Our work assesses the expression stability of the 21 common reference genes in mice LV samples from almost all stages of life cycle. In combination of the expression variabilities of each candidate genes evaluated by GeNorm [19], NormFinder [21], BestKeeper [22], Delta-Ct [23] and RefFinder [24] tools, we propose a set of optimal reference genes reliable for normalization of RT-qPCR data in different specific conditions.

## Methods

### Sample collection

**Fig. 1:**
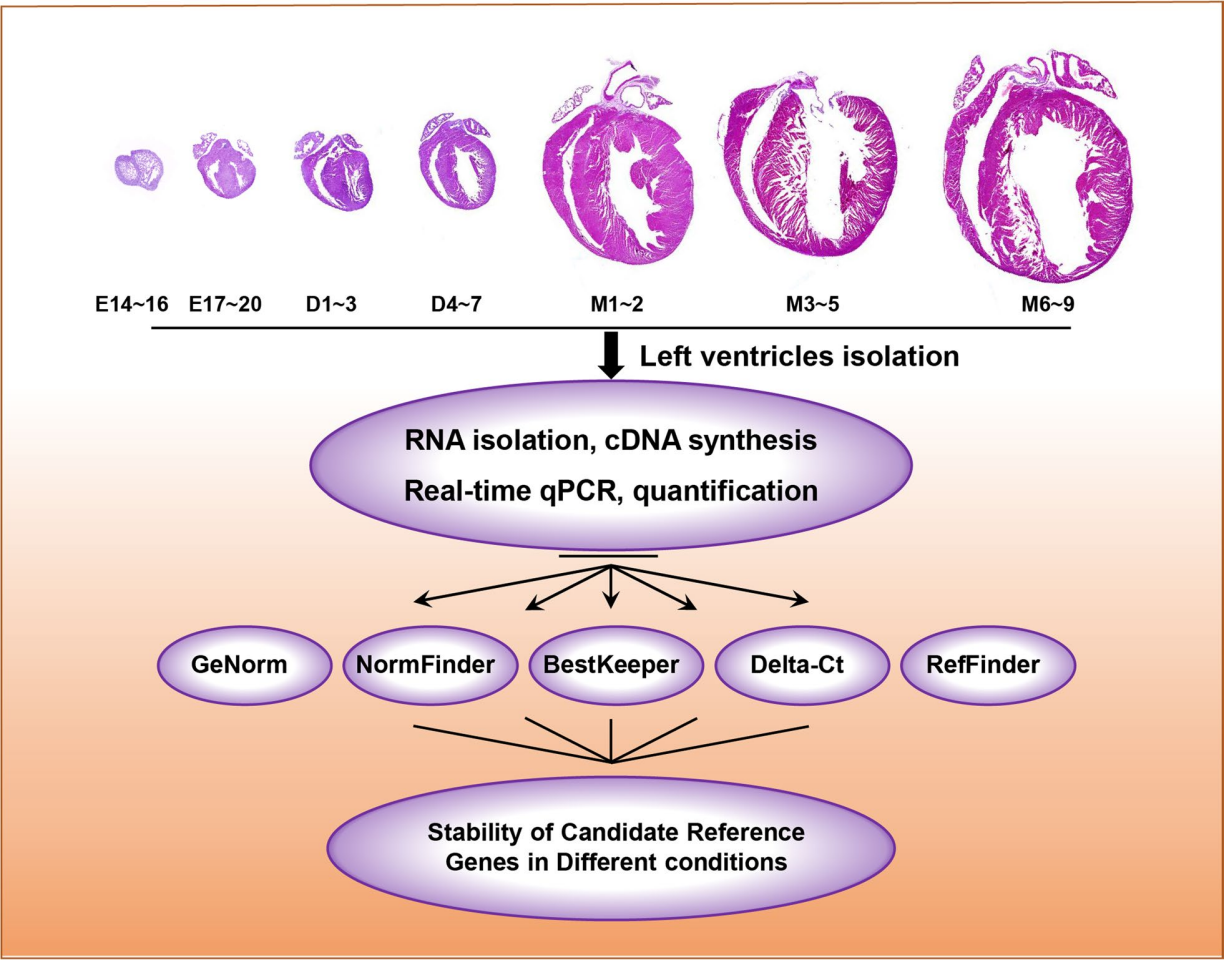
Flow chart of the study design. Illustration of the developmental stages of the mice left ventricle tissues sampled in this study and the short description of the core experiment manipulations. The statistical applications for evaluating the expression stability of reference genes were also shown. E = embryonic day; D = postnatal day; M = postnatal month

The reporting of animal experiments according to the the ARRIVE guidelines (Additional file 1). The C57BL/6 mice were purchased from Vital River Laboratory Animal Technology Co., Ltd (Beijing, China), and maintained in plastic cages at 23 to 25 °C with a 12/12-h light/dark cycle. The experimental protocol was approved by the Animal Care and Use Committee of Fuwai hospital. The animals were mated overnight. Matings were determined by detection of vaginal plugs (taken as day 0.5 of gestation). The mice were euthanized by CO2 inhalation before sample collection. Specifically, mice were placed in a standard mouse cage, and euthanized via CO2 gas asphyxiation with a displacement rate of 20% of the chamber volume per min. Subsequently, cervical dislocations were performed to assure euthanasia. The distribution of samples in each group was as follows: embryonic day 14–16 (n = 4), embryonic day 17–20 (n = 5), postnatal day 1–3 (n = 6), postnatal day 4–7 (n = 5), postnatal month 1 ~ 2 (n = 5), postnatal month 3–5 (n = 7), postnatal month 6–9 (n = 6) (Fig. 1). LV in embryonic hearts were identified and collected by careful microdissection under stereomicroscope (Leica).

### RNA isolation and cDNA synthesis

RNA was isolated from 30 mg frozen LV tissue. MagNA Lyser Green Beads and the MagNA lyser instrument (Roche, Switzerland) were used for tissue homogenate. Then the standard instructions of Trizol manufacturer (Invitrogen, USA) were carried out for total RNA extraction. The concentration and quality of the extracted RNA were evaluated by NanoDrop2000 (NanoDrop Technology, USA). First strand cDNA was synthesized from 500 ng total RNA by using the Takara Reverse Transcription Kit (Takara, Japan). All samples within this experiment were processed simultaneously to avoid interexperimental variations.

### Selection of candidate reference genes and primer design

In this study, twenty-one widely used housekeeping genes (Table 1) were analyzed to provide a better reference guide for identification of molecular mechanisms underlying cardiac development and maturation. Those candidate genes included the *Actb* (Actin beta), *Gapdh* (Glyceraldehyde-3-phosphate dehydrogenase), *Reep5* (Receptor accessory protein5), (Ribosomal protein L5), *Psmb4* (Proteasome subunit beta 4), *Vcp* (Valosin containing protein), *B2m* (Beta-2-microglobulin), *Gusb* (Glucuronidase beta), *Hmbs* (Hydroxymethylbilane synthase), *Hprt1* (Hypoxanthine phosphoribosyltransferase 1), Ipo8 (Importin 8), *Pgk1* (Phosphoglycerate kinase 1), *Polr2a* (RNA polymerase II subunit A), *Ppia* (Peptidylprolyl isomerase A), *Rplp0* (Ribosomal protein lateral stalk subunit P0), *Tbp* (TATA box binding protein), *Tfrc* (Transferrin receptor), *Ubc* (Ubiquitin C), *Ywhaz* (14–3-3 protein zeta), *18S* (eukaryotic 18S ribosomal RNA) and *Sdha* (succinate dehydrogenase complex flavoprotein subunit A). The NCBI primer designing tool was used to generate the RT-qPCR primers sequences (Table 1).

**Table 1:** Candidate reference genes and primer sequences

Gene symbol	Gene Name	GenBank Accession	5'-Primer Sequences (Forward/Reverse)-3'	Product size (bp)
Actb	Actin beta	NM_007393	GGCTGTATTCCCCTCCATCG / CCAGTTGGTAACAATGCCATGT	154
Gapdh	Glyceraldehyde-3-phosphate dehydrogenase	NM_001289726	AGGTCGGTGTGAACGGATTTG / TGTAGACCATGTAGTTGAGGTCA	123
Reep5	Receptor accessory protein 5	NM_007874	GGTTCCTGCACGAGAAGAACT / GAGAGAGGCTCCATAACCGAA	140
Rpl5	Ribosomal protein L5	NM_016980	TTGGTGATCCAGGACAAGAATAA / GCACAGACGATCATATCCCC	125
Psmb4	Proteasome subunit beta 4	NM_008945	ATGGAAGCGTTTTGGGAGTCA / GTTCTGGGTCCGAGTGATGG	144
Vcp	Valosin containing protein	NM_009503	GCTTGTAAACTGGCCATTCG / GATCTCAGGCACTGGATCGT	114
B2m	Beta-2-microglobulin	NM_009735	TTCTGGTGCTTGTCTCACTGA / CAGTATGTTCGGCTTCCCATTC	104
Gusb	Glucuronidase beta	NM_010368	GGCTGGTGACCTACTGGATTT / GGCACTGGGAACCTGAAGT	131
Hmbs	Hydroxymethylbilane synthase	NM_001110251	AAGGGCTTTTCTGAGGCACC / AGTTGCCCATCTTTCATCACTG	78
Hprt1	Hypoxanthine phosphoribosyltransferase 1	NM_013556	GGTTAAGCAGTACAGCCCCA / GGCCTGTATCCAACACTTCG	81
Ipo8	Importin 8	NM_001081113	ACGTGACAGTAGATACCAACGC / GCATAGCACTCGGCATCTTCT	115
Pgk1	Phosphoglycerate kinase 1	NM_008828	ATGTCGCTTTCCAACAAGCTG / GCTCCATTGTCCAAGCAGAAT	164
Polr2a	RNA polymerase II subunit A	NM_001291068	AAATACCCAGAAACAACGGAGG / CCAGTCCGCTCAATCACCC	83
Ppia	Peptidylprolyl isomerase A	NM_008907	GAGCTGTTTGCAGACAAAGTTC / CCCTGGCACATGAATCCTGG	125
Rplp0	Ribosomal protein lateral stalk subunit P0	NM_007475	AGATTCGGGATATGCTGTTGGC / TCGGGTCCTAGACCAGTGTTC	109
Tbp	TATA box binding protein	NM_013684	GTGGGGAGCTGTGATGTGA / TCCAGGAAATAATTCTGGCTCA	96
Tfrc	Transferrin receptor	NM_011638	GTTTCTGCCAGCCCCTTATTAT / GCAAGGAAAGGATATGCAGCA	152
Ubc	Ubiquitin C	NM_019639	GAGGTGGCATGCAGATCTTT / CCCTCCTTGTCCTGGATCTT	112
Ywhaz	Tyrosine 3-monooxygenase/tryptophan 5-monooxygenase activation protein zeta	NM_011740	GAAAAGTTCTTGATCCCCAATGC / TGTGACTGGTCCACAATTCCTT	134
18S	eukaryotic 18S ribosomal RNA	NR_003278	CTCAACACGGGAAACCTCAC / CGCTCCACCAACTAAGAACG	110
Sdha	succinate dehydrogenase complex flavoprotein subunit A	NM_023281	GGAACACTCCAAAAACAGACCT / CCACCACTGGGTATTGAGTAGAA	106

### RT-qPCR

RT-qPCR was performed on the Viia7 384-well block Real Time PCR System (Applied Biosystems). Each 10 µl reaction mixture contained the 5 µl 2 × SYBR Green Real-Time PCR Master Mix reaction mixture, 0.4 µL of each primer (10 µM), 2 μl cDNA (2.5 ng/μl) and 2.2 µL double-distilled water. The thermal cycling program: 95 °C for 10 min, followed by 40 cycles of 15 s at 95  °C and 1 min at 60 °C. Each sample was performed in three technical replicates.

### Evaluation of expression stability

The data analysts were blind to the experimental groupings. The gene expression stability was evaluated by analyzing the raw cycle threshold (Ct) values in four independent statistical applications: GeNorm, Normfinder, BestKeeper and Delta-Ct method. And a consensual analysis (RefFinder) was performed to make a comprehensive variability score for each reference gene.

These methods (GeNorm, NormFinder, and Delta-Ct) were based on similar principles. Take GeNorm for example, the candidate reference genes were ranked by an expression stability measurement called M-value which is based on overall pairwise comparisons with all the other reference genes. The stability value (M-value) is negatively correlated to gene expression stability. But the stability value derived from BestKeeper was based on the coefficient of variation (CV) and standard deviation (SD) values. So, a consensual statistical analysis of the variabilities of the housekeeping genes was needed. Based on previous studies [24], we employed the RefFinder analysis to obtain a comprehensive evaluation of candidate reference genes by integrating all above-mentioned four algorithm results. The overall rank order of the stable reference genes is shown in Additional file 2: Table S1 after comparisons.

### Statistical analysis

Continuous variables are expressed as the mean ± SD without special instructions. One-way analysis of variance (ANOVA) or Kruskal–Wallis test were performed to evaluate the difference among three groups or more. Differences with a 2-tail P-value < 0.05 were considered statistically significant. All statistical analyses were performed using SPSS Statistics, version 23.0 (IBM Corp, Armonk, NY), and graphs were generated using GraphPad Prism 7 (GraphPad Software Inc., CA).

## Results

### Expression characteristics of the candidate reference genes

e showed the full names and corresponding GeneBank accession numbers of the 21 candidate housekeeping genes in Table 1. Table 2 summarized the total RNA concentration obtained at different stages of heart development, which ranged from 61.08 to 83.38 ng/μl. The extracted RNA quality was assessed by Nanodrop 2000 Spectrophotometer. Both ratios (260 nm/280 nm ratio and 260 nm/230 nm ratio) were close to 2.0, indicating the high quality of the extracted RNA samples.

**Table 2:** The quality of RNA samples isolated from left ventricles

	N	RNA concentration (ng/ul)	A260 (Abs)	260 nm/280 nm ratio		260 nm/230 nm ratio	
				Mean	SD	Mean	SD
E 14 ~ 16	4	67.65 ± 2.92	1.70 ± 0.07	1.97	0.03	2.13	0.09
E 17–20	5	66.98 ± 2.40	1.67 ± 0.06	1.99	0.03	2.08	0.13
D 1 ~ 3	6	61.08 ± 4.56	1.53 ± 0.11	2.02	0.01	2.09	0.07
D 4 ~ 7	5	69.12 ± 9.59	1.73 ± 0.24	2.03	0.01	2.05	0.14
M 1 ~ 2	5	83.38 ± 22.58	2.08 ± 0.56	2.05	0.02	2.21	0.05
M 3 ~ 5	7	65.57 ± 4.78	1.64 ± 0.12	2.08	0.02	1.97	0.17
M 6 ~ 9	6	66.17 ± 3.67	1.65 ± 0.09	2.06	0.02	2.16	0.08

E = embryonic day; D = postnatal day; M = postnatal month; N = sample size; SD = standard deviation

**Fig. 2:**
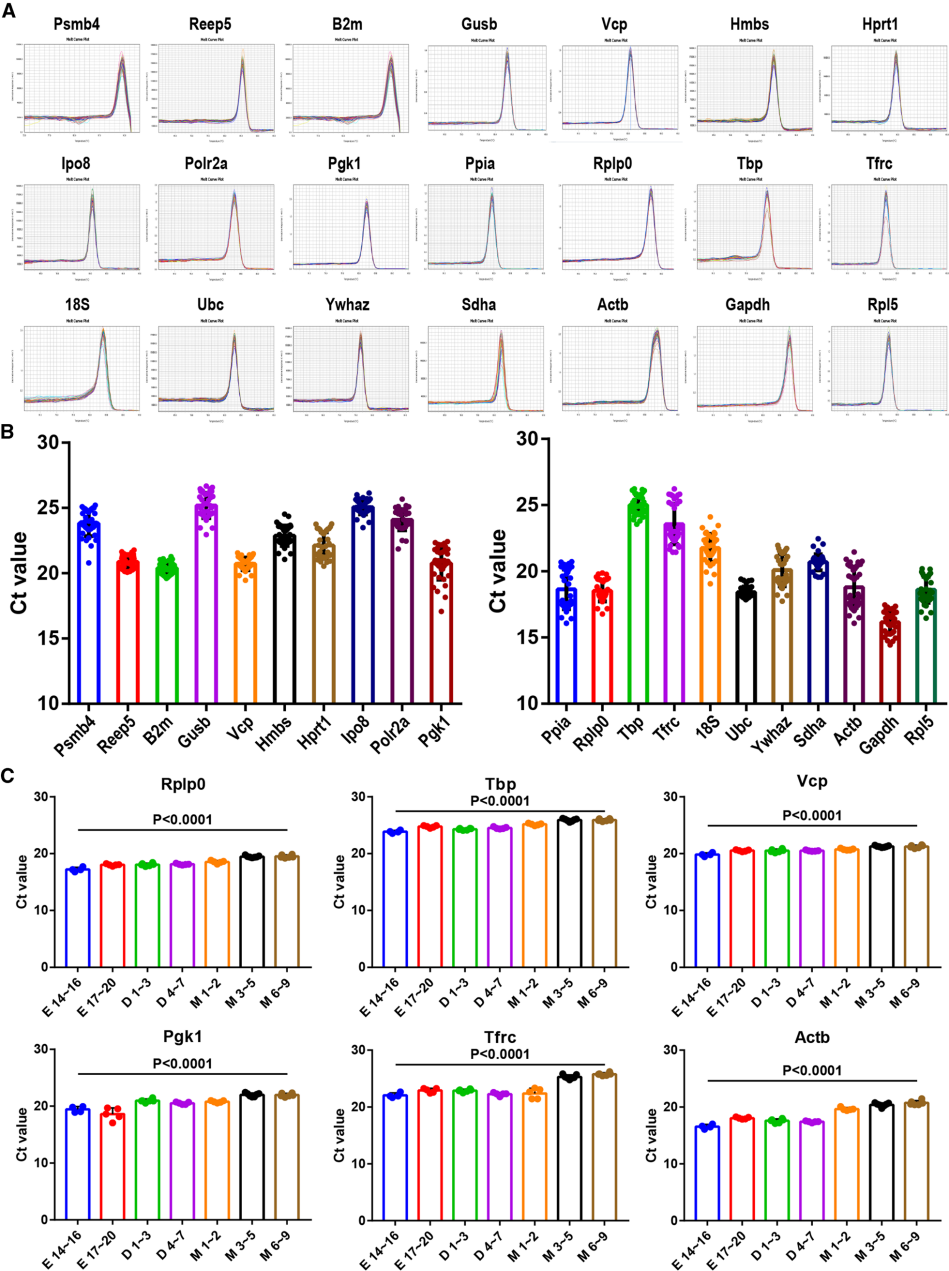
Overall abundance of the reference genes during development of mouse heart. (**A**) The melting curve assays of the 21 candidate reference genes across all samples showed the single peak, indicating the RT-qPCR amplification had good specificity; (**B**) Expression levels of candidate reference gene expression in all heart samples. (**C**) The expression variabilities of 6 housekeeping genes at 7 different developmental stages. The plots represented the gene expression level of each candidate reference gene in heart samples (n = 38). Values are given as cycle threshold values (Ct values), mean and standard deviation of the Ct values were indicated in the plot

The primer information was shown in Table 1. The melting curves for all candidate genes exhibited single peaks, indicating decent specificity (Fig. 2A). The Ct-values obtained by qPCR were used to quantify the gene expression levels. The general abundance and variation in candidate reference genes were illustrated in Fig. 2B–C and Additional file 3: Figure S1. Ct values ranged from 16.16 (*Gapdh*) to 25.19 (*Gusb*). As shown in Fig. 2C and Additional file 3: Figure S1, the patterns of gene expression of these reference genes were stable at same developmental stage. This suggested that the variability mainly from the different developmental stages, not from the individual differences at the same stage. So, the conventional normalization genes may still be fine for comparison of samples at the same developmental stage. We found that some genes have more stable expression across different developmental stages (such as *Rplp0*, *Tbp*, *Vcp*), than others, e.g., *Pgk1, Tfrc, Actb* (Additional file 2: Table S1).

**Fig. 3:**
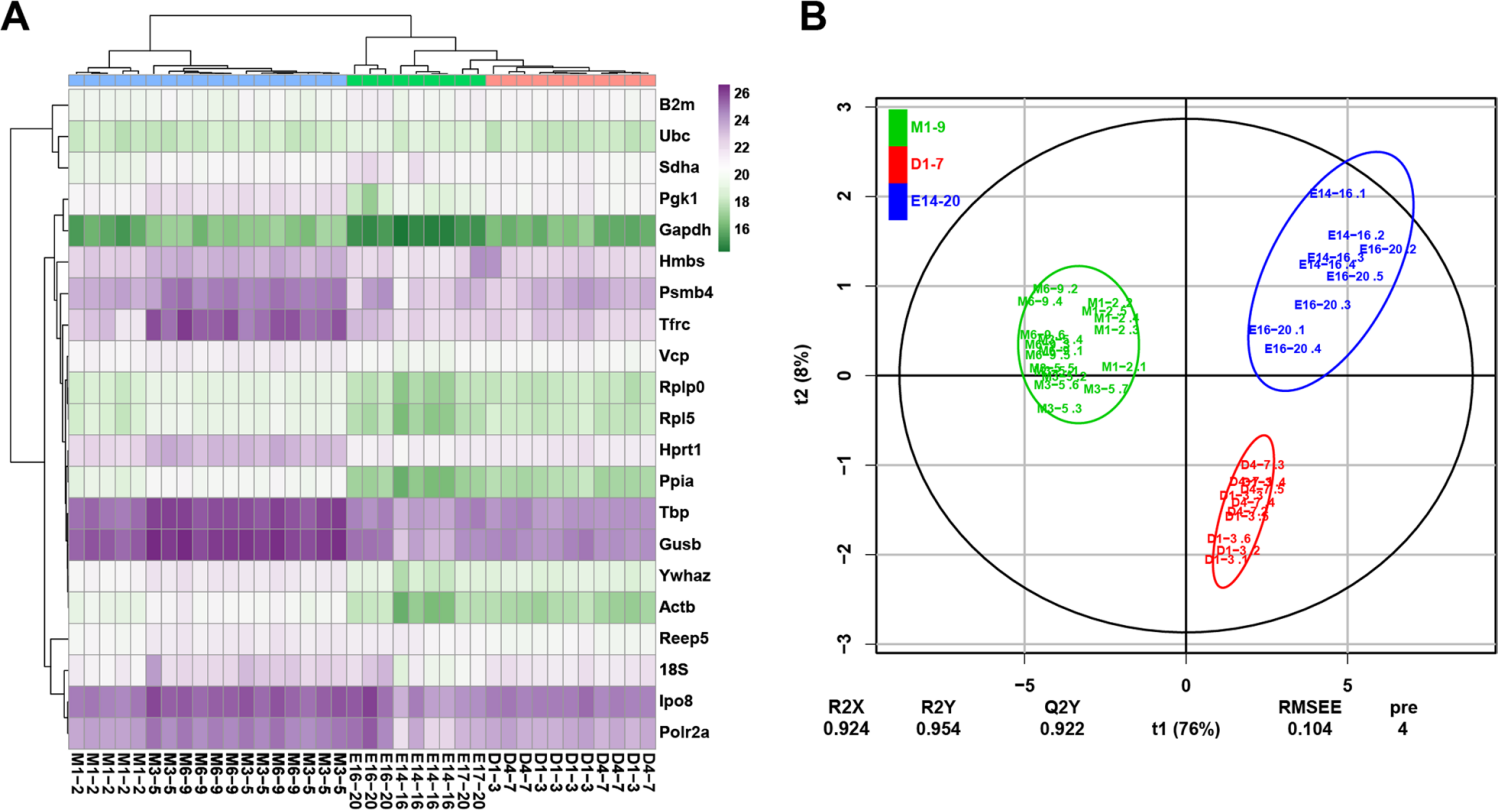
Clustering analysis of the candidate reference genes expression. (**A**) Heatmap of hierarchical cluster analysis of samples performed on the profiles of 21 candidate reference genes to depict the similarity of gene profiles; (**B**) Supervised Orthogonal partial least squared-discriminant (OPLS-DA) score plot. Three phases can be distinguished in the gene expression features, that are (1) embryo stage, (2) first 7 days after birth, (3) 1 to 9 months after birth

For more efficient analysis of the expression pattern of these genes between the different development phases, we used the hierarchical cluster analysis (HCA) [25] and orthogonal projections to latent structures—discriminant analysis (OPLS-DA) [26] to globally visualize the expression classifications. The gene expression features could be divided into three periods, that are (A) embryo stage, (B) first 7 days after birth, (C) 1 to 9 months after birth (Fig. 3).

### Stability of candidate reference genes

**Fig. 4:**
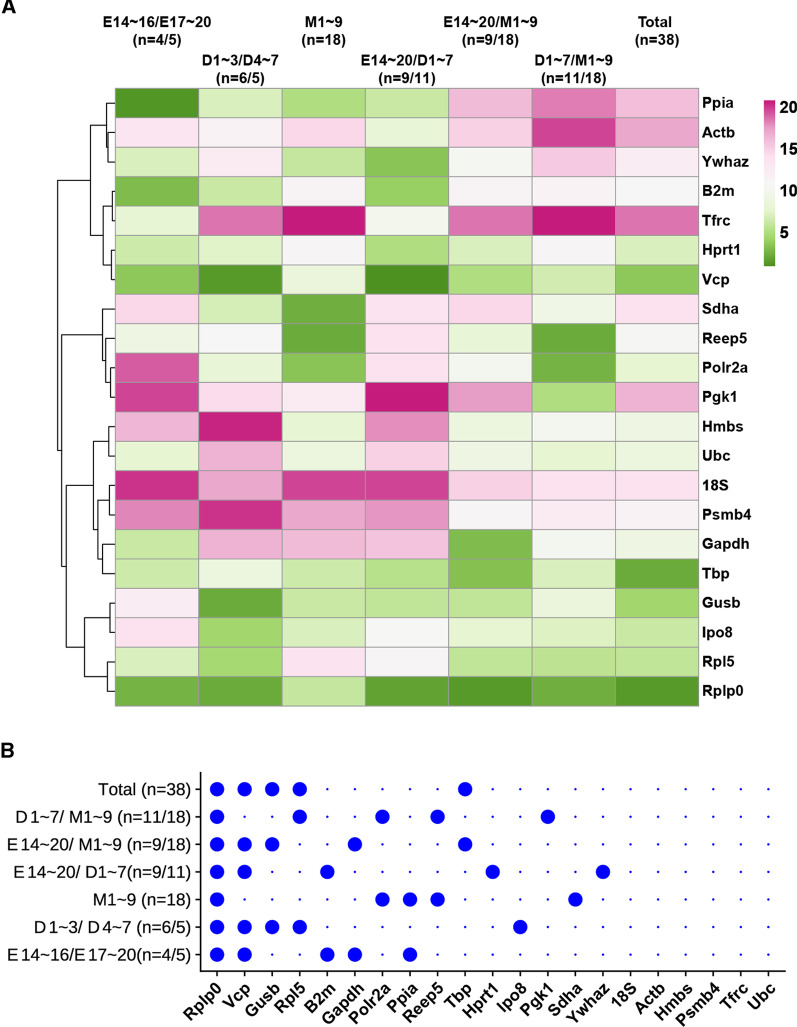
Stability of candidate reference genes. (**A)** Heatmap to illustrate the gene expression stability of 21 candidate reference genes in each dataset with different condition. Column labels: Numbers at the right of the label are “stability value”, which is inversely correlated to gene expression stability. The darker the green the stronger the gene expression stability, the darker the red, the weaker stability; **(B)** Dot plot graph to show the optimal reference genes in each condition

To assess the stability of gene expression, five different tools were used for each specific condition: GeNorm, NormFinder, Delta-Ct, BestKeeper, and RefFinder. Additional file 2: Table S1 demonstrated the results of the expression variabilities of the 21 housekeeping genes analyzed by different statistical methods. The heatmap summarized the stability of candidate reference genes expression at different conditions (Fig. 4A). And the dot plot graph showed the top 5 optimal reference genes in each condition (Fig. 4B). *Rplp0, Tbp, Vcp, Gusb*, and *Rpl5* should be the most suitable gene set out of the 21 reference genes for normalizing gene expression data throughout prenatal to postnatal periods of cardiac development. During the embryonic developmental periods of LV, *Ppia, Rplp0, B2m, Vcp*, and *Gapdh* were selected as the optimal reference genes. However, *Ppia* and *Gapdh* were not recommended for comparison studies of embryonic and neonatal hearts (E14-20 VS. D1-7), while *Vcp, Rplp0*, *Ywhaz, B2m,* and *Hprt*1 are better choices (Additional file 2: Table S1, Fig. 4). Regarding the gene expression stability in LV at embryonic (E14-20) and postnatal maturation stages (M1-9), the results indicated that *Rplp0, Gapdh, Tbp, Vcp, Gusb* should be the appropriate reference genes. *Reep5, Rplp0, Polr2a, Pgk1, Rpl5* constituted the best set of reference genes for comparison of the early postnatal (D1-7) and postnatal maturation stages (M1-9) (Additional file 2: Table S1, Fig. 4). Our results also showed that 6 genes, including *18S, Hmbs, Ubc, Psmb4, Tfrc* and *Act,* are not recommended to be used for normalization of RT-qPCR data in developmental murine hearts while *Rplp0* might serve as an optimal reference gene in gene expression analysis.

### Gene expression levels normalized by different reference gene

**Fig. 5:**
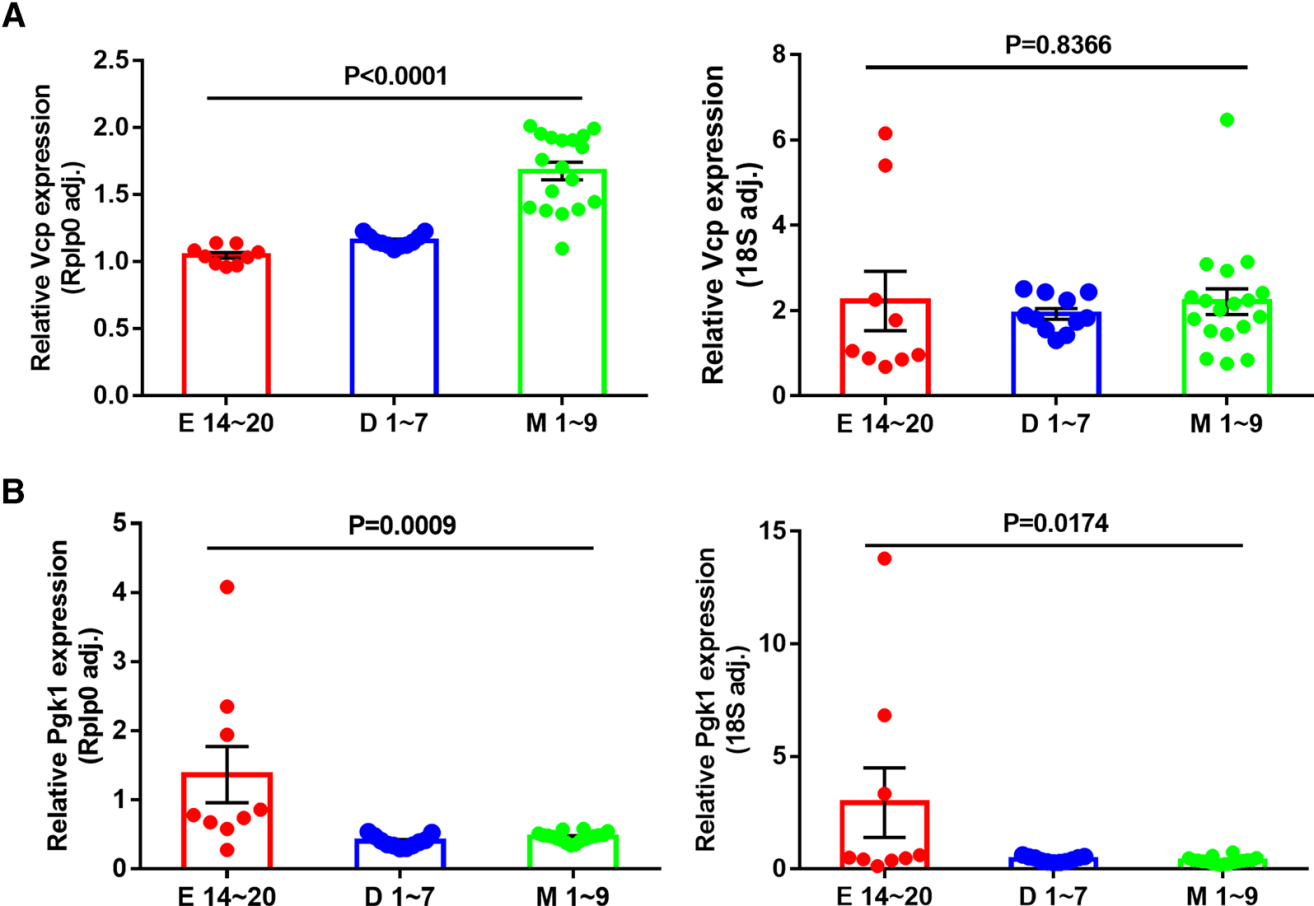
Relative expression of two target genes in different heart developmental periods. (**A)** *Vcp* expression detection normalized by reference gene *Rplp0* or *18S*; **(B)** *Pgk1* expression detection normalized by reference gene *Rplp0* or *18S*. Values are given as relative gene expression level, mean and standard deviation of the values were indicated in the plot

We further verified the results by using different internal reference genes to normalize the RT-qPCR data. The most and least stable reference gene (*Rplp0* and *18S*) were used for RT-qPCR data analysis with the same sample. As shown in Fig. 5, the selection of proper internal reference gene posed a profound effect on assessment of target gene expression levels. Obvious difference in *Vcp* expression was observed among the groups normalized by *Rplp0.* However, no significant difference in the target gene expression was observed when using *18S* as the reference gene (Fig. 5A). Similar trends were also found when using *Pgk1* as the target gene: Compared with the results with reference to *Rplp0*, the significance of difference among the groups is significantly reduced with *18S* as the reference gene (Fig. 5B).

## Discussions

Previous literatures pointed out that there is no perfect reference gene appropriate to all conditions in RT-qPCR experiments [16]. We presented a detailed reference gene selection scheme for RT-qPCR studies in cardiac development and maturation.

The RT-qPCR is the most common and useful method for assessing the gene expression characteristics. Selecting an appropriate reference gene for normalization in RT-qPCR experiments is important to reduce the effect of sample heterogeneity and provide accurate results [11, 27]. The mechanism of heart development, which was not completely elucidated, was often studied using transcriptomics. Thus, it is crucial to identify the stable reference genes throughout prenatal to postnatal periods of cardiac development. The previous studies involving reference genes primarily based on myocardial tissues from adult mice or whole hearts [28]. Another limitation of previous studies is that only a limited number of candidate reference genes and a small number of samples were included [20, 28, 29]. In this study, we investigated the gene expressions of 21 candidate housekeeping genes at 7 different developmental stages, which cover almost all major stages in the life cycle. We therefore believe the results from our study is likely to be more applicable and comprehensive.

Previous investigators demonstrated that expression of some candidate reference genes significantly varied in different conditions [20]. For example, the expression levels of gene *18S, Actb* and *Gapdh* held considerable alterations upon different developmental stages and experimental conditions [30, 31]. It is worth mentioning that the expression of *Gapdh* and *Actb* apparently fluctuated even during the normal development of heart. In our study, *18S* and *Actb* did not hold a sufficiently stable expression pattern. Thus, they might not be ideal reference genes in LV development or maturation-related studies. *Gapdh* may be redeemed as a better candidate reference gene only under specific conditions such as studying LV at embryonic stages or comparing embryonic (E14-20) VS. postnatal maturation stages (M1-9). There is a congruence between this data and our transcriptomic data of human heart samples at different developmental stages (in-house data), which suggests *Actb* and *18S* were unsuitable to serve as reference genes in heart development studies. Our results demonstrated that *Rplp0* is more stable in expression. Rplp0 protein, as a component of the 60S subunit, is involved in the regulatory process of protein synthesis [32]. It was the only reference gene that could be applied to all subgroup analysis in our study. Based on the results, we propose that *Rplp0* is an optimal reference gene in mice LV development or maturation.

Gene expression is dynamic during heart development from a linear tube to four-chambered heart. This complexity further increases when disease conditions or injury models are included. Trond Brattelid et al. evaluated the optimal reference genes in mouse myocardium from different developmental stages (fetal and neonatal period) and heart failure condition [33]. Similar to our results, *Gapdh* held a wide variation in expression at different developmental stages. They also found *Rpl4* and *Rpl32* were most stable from neonatal to adult myocardium[33]. While we found that *Rplp0* gives the best performance. These genes all belong to ribosomal protein family and are parts of ribosomal 60 s subunit. In the condition of post-infarction heart failure, *Polr2a* was the better reference gene [33]. Likewise, we also find the *Polr2a* is a stable reference gene for comparison of the early postnatal (D1-7) and postnatal maturation stages (M1-9). Adrián Ruiz-Villalba et al. investigated the expression stabilities of reference genes in different subsets of mouse myocardium from cardiac development to pathology [28]. *Ppia* was recommended for normalization in comparison studies of prenatal hearts, which was the same as the results in our study. It is interesting to note that the optimal reference genes for analysis in the group “adult” or the group “adult pathologies” are the same [28]. Bert R Everaert et al. showed *Gapdh*, *Actb*, and *B2m* might not suitable for application in myocardial infarction studies [31]. These same genes were also not recommended in our study. It demonstrated *Hprt*, *Rpl13a* and *Tpt1* should be the most suitable gene set for normalizing in a mouse myocardial infarction model [31].These results are markedly different from ours, which illustrates the expression stabilities of reference genes in pathological state significantly differ from the normal physiological state. These identified reference genes should be regarded as good candidates in RT-qPCR experiment, but the expression stability in each particular experimental setting is still recommended to validate.

Moreover, different reference genes may lead to completely different results when analyzing the expression of target genes. Unsuitable reference gene could lead to biased results and even wrong conclusions. This also emphasizes the importance of selecting the optimal reference gene when studying the transcriptomic signatures in heart development or maturation process. And the research findings from normal heart development and maturation are essential foundations for various pathological conditions-induced cardiac damage. Therefore, the need for validated stable reference genes in normal cardiac development and maturation should be emphasized. Nevertheless, we have to acknowledged that our study is limited by the fact that we did not take into account the other developmental disease conditions or injury models. In this respect, additional investigations are required.

## Conclusions

Our study provides the expression stability of the commonly reference genes in process of LV development and maturation. We propose a set of optimal reference genes under different conditions and suggest *Rplp0* could serve as a stable reference gene of LV tissue across different developmental stages. Our findings may be helpful in future studies for investigating the gene expression patterns of mammalian LV development.

## Supplementary Information

**Additional file 1**. The ARRIVE checklist. The reporting of animal experiments according to the the ARRIVE guidelines (http://www.nc3rs.org.uk/page.asp?id=1357).

**Additional file 2: Table S1**. Reference gene expression variability and rankings. Demonstrating the results of the different statistical methods (GeNorm, NormFinder, Delta-Ct, BestKeeper and RefFinder) for analyzing the temporality expression variabilities of 21 housekeeping genes in the different comparisons among groups addressed.

**Additional file 3: Figure S1**. The expression variabilities of 15 housekeeping genes at 7 different developmental stages were plotted with bar and scatter graphs. Values are given as raw cycle threshold (Ct) values, mean and standard deviation of the Ct values were indicated in the plot.

## Data Availability

All data generated or analysed during this study are included in this published article and any supplementary material. The datasets generated and/or analysed during the current study are available in https://doi.org/10.6084/m9.figshare.15407625.v1.

## Abbreviations

RT-qPCR: Real-time quantitative polymerase chain reaction
LV: Left ventricle
Ct: Cycle threshold
SD: Standard deviation
E: Embryonic day
D: Postnatal day
M: Postnatal month
