# Supplementary material for: Identification of reference genes for gene expression studies among different developmental stages of murine hearts
Source: BMC Dev Biol. 2021 Sep 8;21:13. doi: 10.1186/s12861-021-00244-6 (PMC8425138; doi:10.1186/s12861-021-00244-6)
Supplement: Supplementary file 1 — Additional file 1. The ARRIVE checklist. The reporting of animal experiments according to the the ARRIVE guidelines (http://www.nc3rs.org.uk/page.asp?id=1357). [file 12861_2021_244_MOESM1_ESM.docx]

**The ARRIVE checklist**

**1. Study design**

In this study, mice left ventricles were collected at different embryonic and postnatal stages (embryonic day 14~16 (n=4), embryonic day 17~20 (n=5), postnatal day 1~3 (n=6), postnatal day 4~7 (n=5), postnatal month 1~2 (n=5), postnatal month 3~5 (n=7), postnatal month 6~9 (n=6)). We investigated the gene expressions of common 21 candidate housekeeping genes in mice LV from 7 different developmental stages. A number of methods, such as GeNorm, NormFinder, BestKeeper, Delta-Ct and RefFinder method, were used to identify a set of optimal reference genes in different developmental stages of mice LV. (**Methods, paragraph 1,5**)

**2. Sample size**

The exact number of experimental mice in each group were showed in **Methods, paragraph 1**.

**3. Inclusion and exclusion criteria**

Normal mice (male C57BL/6) of specified ages (E14~16, E17~20, D1~3, D4~7, M1~2, M3~5, M6~9) were included. In case of structural or functional abnormality of the heart, participants were excluded. (**Methods, paragraph 1**)

**4. Randomization**

N/A

**5. Blinding**

In regard to experimental grouping, the data analysts were blind. (**Methods, paragraph 5**)

**6. Outcome measures**

The gene expression stabilities of common 21 candidate housekeeping genes in mice LV from 7 different developmental stages, were evaluated by analyzing the raw Ct values in the four independent statistical applications. (**Methods, paragraph 5**)

**7. Statistical methods**

The gene expression stability was evaluated by analyzing the raw Ct values in the four independent statistical applications: GeNorm, Normfinder, BestKeeper and Delta-Ct method. And we also performed a consensual analysis by RefFinder to make a comprehensive variability score for each reference gene. (**Methods, paragraph 5**)

Continuous variables are expressed as the mean ± the standard deviation (SD) without special instructions. A one-way analysis of variance (ANOVA) was performed to calculate whether there is statistically different among groups. Differences with a P-value <0.05 were considered statistically significant. All statistical analyses were performed using SPSS Statistics, version 23.0 (IBM Corp, Armonk, NY), and graphs were generated using GraphPad Prism 7 (GraphPad Software Inc., CA). (**Methods, paragraph 6**)

**8. Experimental animals**

The C57BL/6 mice were purchased from Vital River Laboratory Animal Technology Co., Ltd (Beijing, China). Mice left ventricles were collected at different embryonic and postnatal stages (embryonic day 14~16 (n=4), embryonic day 17~20 (n=5), postnatal day 1~3 (n=6), postnatal day 4~7 (n=5), postnatal month 1~2 (n=5), postnatal month 3~5 (n=7), postnatal month 6~9 (n=6)). LV in embryonic hearts were identified and collected by careful microdissection under stereomicroscope (Leica) using sharpened microdissection scissors. (**Methods, paragraph 1**)

**9. Experimental procedures**

① RNA isolation and cDNA synthesis

Each RNA isolation was performed from 30 mg of the frozen LV tissue. MagNA Lyser Green Beads (Roche, Switzerland) containing TRIzol (Invitrogen, USA) and the MagNA lyser instrument (Roche, Switzerland) were used for tissue homogenate. Then the standard instructions of Trizol manufacturer were carried out for total RNA extraction. The concentration and quality were evaluated by NanoDrop2000 (NanoDrop Technology, USA). For each sample, First Strand cDNA was synthesized from 500ng total RNA by using the Takara Reverse Transcription Kit (Takara, Japan). All samples within this experiment were processed simultaneously to avoid interexperimental variations. (**Methods, paragraph 2**)

② Selection of candidate reference genes and Primer design

In this study, the 21 housekeeping genes (Table 1), which were commonly used as reference genes in previous cardiac studies, were analyzed to provide a better reference guide for identification of molecular mechanisms underlying cardiac development and maturation. According to the published mouse sequences, the NCBI primer designing tool was used to generate the RT-qPCR primers sequences (Table 1). (**Methods, paragraph 3**)

③ RT-qPCR

RT-qPCR was performed in 384 well plates using the Viia7 Real Time PCR System (Applied Biosystems). Each 10 µl reaction mixture contained the 5 µl 2× SYBR Green Real-Time PCR Master Mix reaction mixture, 0.4 µL of each primer (10 µM), 2μl cDNA (2.5 ng/μl) and 2.2 µL double-distilled water. The thermal cycling program: 95℃ for 10 min, followed by 40 cycles of 15 s at 95℃ and 1 min at 60℃. Each sample was performed in three technical replicates and the resulting Ct values averaged. (**Methods, paragraph 4**)

④ Evaluation of Expression Stability

The gene expression stability was evaluated by analyzing the raw Ct values in the four independent statistical applications: GeNorm, Normfinder, BestKeeper and Delta-Ct method. And we also performed a consensual analysis by RefFinder to make a comprehensive variability score for each reference gene. (**Methods, paragraph 5**)

**10. Results**

We summarized these results of the overall gene stability in each dataset with different condition using the heatmap or dot plot graph to illustrate the most suitable 5 reference genes for calculating the gene expression (**Figure 3**).
