# Supplementary material for: Identification of reference genes for gene expression studies among different developmental stages of murine hearts
Source: BMC Dev Biol. 2021 Sep 8;21:13. doi: 10.1186/s12861-021-00244-6 (PMC8425138; doi:10.1186/s12861-021-00244-6)
Supplement: Supplementary file 2 — Additional file 2: Table S1. Reference gene expression variability and rankings. Demonstrating the results of the different statistical methods (GeNorm, NormFinder, Delta-Ct, BestKeeper and RefFinder) for analyzing the temporality expression variabilities of 21 housekeeping genes in the different comparisons among groups addressed. [file 12861_2021_244_MOESM2_ESM.docx]

**Supplementary Table S1. Reference gene expression variability and rankings**

|  | **GeNorm** | |  | **NormFinder** | |  | **BestKeeper** | |  | **Delta CT** | |  | **RefFinder** | |
| --- | --- | --- | --- | --- | --- | --- | --- | --- | --- | --- | --- | --- | --- | --- |
|  | **Gene name** | **M value** |  | **Gene name** | **Stability value** |  | **Gene name** | **std dev** |  | **Gene name** | **STDEV** |  | **Gene name** | **Geomean of ranking values** |
| **Total (n=38)** | |  |  |  |  |  |  |  |  |  |  |  |  |  |
|  | Rplp0 | 0.221 |  | Rplp0 | 0.044 |  | Ubc | 0.33 |  | Rplp0 | 0.61 |  | **Rplp0** | 1.78 |
|  | Tbp | 0.221 |  | Tbp | 0.188 |  | B2m | 0.37 |  | Tbp | 0.64 |  | **Tbp** | 2.45 |
|  | Gusb | 0.268 |  | Vcp | 0.267 |  | Vcp | 0.39 |  | Gusb | 0.66 |  | **Vcp** | 3.83 |
|  | Rpl5 | 0.299 |  | Gusb | 0.277 |  | Sdha | 0.4 |  | Vcp | 0.67 |  | **Gusb** | 4.65 |
|  | Hprt1 | 0.321 |  | Rpl5 | 0.312 |  | Ipo8 | 0.43 |  | Rpl5 | 0.67 |  | **Rpl5** | 6.12 |
|  | Vcp | 0.373 |  | Hprt1 | 0.389 |  | Reep5 | 0.46 |  | Hprt1 | 0.71 |  | Ipo8 | 6.44 |
|  | Ipo8 | 0.429 |  | Ipo8 | 0.405 |  | Hmbs | 0.64 |  | Ipo8 | 0.73 |  | Hprt1 | 7.33 |
|  | Polr2a | 0.457 |  | Polr2a | 0.432 |  | Polr2a | 0.65 |  | Polr2a | 0.75 |  | Polr2a | 8 |
|  | Gapdh | 0.486 |  | Gapdh | 0.476 |  | Tbp | 0.66 |  | Gapdh | 0.78 |  | Ubc | 8.98 |
|  | Hmbs | 0.51 |  | Hmbs | 0.502 |  | Rplp0 | 0.68 |  | Ywhaz | 0.79 |  | Hmbs | 9.37 |
|  | Ywhaz | 0.533 |  | Psmb4 | 0.52 |  | Gapdh | 0.69 |  | Hmbs | 0.8 |  | Gapdh | 9.46 |
|  | Psmb4 | 0.552 |  | Ywhaz | 0.568 |  | Psmb4 | 0.77 |  | Psmb4 | 0.81 |  | Reep5 | 10.72 |
|  | Reep5 | 0.58 |  | Reep5 | 0.57 |  | Gusb | 0.81 |  | Reep5 | 0.83 |  | B2m | 11.1 |
|  | 18S | 0.61 |  | 18S | 0.683 |  | Rpl5 | 0.82 |  | 18S | 0.92 |  | Psmb4 | 11.74 |
|  | Ppia | 0.651 |  | Ppia | 0.882 |  | 18S | 0.85 |  | Ppia | 1.01 |  | Ywhaz | 12.42 |
|  | Actb | 0.682 |  | Pgk1 | 0.902 |  | Hprt1 | 0.91 |  | Actb | 1.04 |  | Sdha | 13.87 |
|  | Pgk1 | 0.716 |  | Actb | 0.918 |  | Pgk1 | 0.93 |  | Pgk1 | 1.07 |  | 18S | 14.24 |
|  | Tfrc | 0.748 |  | Tfrc | 0.932 |  | Ywhaz | 1.11 |  | Ubc | 1.07 |  | Ppia | 16.12 |
|  | Ubc | 0.79 |  | Ubc | 0.946 |  | Tfrc | 1.34 |  | B2m | 1.09 |  | Pgk1 | 16.74 |
|  | B2m | 0.823 |  | B2m | 0.973 |  | Ppia | 1.4 |  | Tfrc | 1.1 |  | Actb | 17.39 |
|  | Sdha | 0.851 |  | Sdha | 1.004 |  | Actb | 1.42 |  | Sdha | 1.12 |  | Tfrc | 18.73 |
| **E 14~16/E 17~20(n=4/5)** | |  |  |  |  |  |  |  |  |  |  |  |  |  |
|  | Ppia | 0.064 |  | Ppia | 0.032 |  | Hprt1 | 0.24 |  | Ppia | 0.46 |  | **Ppia** | 1.57 |
|  | Rplp0 | 0.064 |  | B2m | 0.039 |  | Reep5 | 0.26 |  | B2m | 0.46 |  | **Rplp0** | 2.82 |
|  | Vcp | 0.081 |  | Rplp0 | 0.099 |  | Gapdh | 0.27 |  | Rplp0 | 0.47 |  | **B2m** | 3.36 |
|  | B2m | 0.093 |  | Vcp | 0.13 |  | Vcp | 0.35 |  | Vcp | 0.47 |  | **Vcp** | 3.94 |
|  | Tbp | 0.109 |  | Ywhaz | 0.138 |  | Ubc | 0.35 |  | Tbp | 0.5 |  | **Gapdh** | 6.42 |
|  | Rpl5 | 0.12 |  | Tfrc | 0.161 |  | Ppia | 0.4 |  | Ywhaz | 0.5 |  | Hprt1 | 6.57 |
|  | Gapdh | 0.14 |  | Rpl5 | 0.185 |  | Rplp0 | 0.41 |  | Rpl5 | 0.5 |  | Tbp | 6.69 |
|  | Ywhaz | 0.174 |  | Tbp | 0.186 |  | B2m | 0.44 |  | Tfrc | 0.51 |  | Rpl5 | 7.17 |
|  | Tfrc | 0.206 |  | Gapdh | 0.258 |  | Rpl5 | 0.44 |  | Gapdh | 0.52 |  | Ywhaz | 7.33 |
|  | Ubc | 0.233 |  | Ubc | 0.263 |  | Tbp | 0.45 |  | Ubc | 0.55 |  | Ubc | 7.95 |
|  | Hprt1 | 0.254 |  | Gusb | 0.317 |  | Tfrc | 0.47 |  | Gusb | 0.57 |  | Tfrc | 8.3 |
|  | Gusb | 0.284 |  | Actb | 0.334 |  | Ywhaz | 0.57 |  | Actb | 0.59 |  | Reep5 | 9.36 |
|  | Actb | 0.303 |  | Hprt1 | 0.394 |  | Sdha | 0.62 |  | Hprt1 | 0.61 |  | Gusb | 12.35 |
|  | Ipo8 | 0.341 |  | Ipo8 | 0.487 |  | Ipo8 | 0.67 |  | Ipo8 | 0.68 |  | Actb | 13.55 |
|  | Reep5 | 0.378 |  | Sdha | 0.573 |  | Hmbs | 0.68 |  | Sdha | 0.75 |  | Ipo8 | 14 |
|  | Sdha | 0.409 |  | Reep5 | 0.593 |  | Gusb | 0.7 |  | Reep5 | 0.76 |  | Sdha | 14.71 |
|  | Hmbs | 0.462 |  | Hmbs | 0.806 |  | Pgk1 | 0.7 |  | Hmbs | 0.94 |  | Hmbs | 16.48 |
|  | Psmb4 | 0.51 |  | Psmb4 | 0.808 |  | Actb | 0.74 |  | Psmb4 | 0.95 |  | Psmb4 | 18.24 |
|  | Polr2a | 0.552 |  | Polr2a | 0.857 |  | Psmb4 | 0.85 |  | Polr2a | 0.95 |  | Polr2a | 19.25 |
|  | 18S | 0.605 |  | 18S | 1.063 |  | Polr2a | 1.01 |  | 18S | 1.14 |  | Pgk1 | 19.92 |
|  | Pgk1 | 0.68 |  | Pgk1 | 1.351 |  | 18S | 1.19 |  | Pgk1 | 1.4 |  | 18S | 20.25 |
| **E 14~20/ D1~7(n=9/11)** | |  |  |  |  |  |  |  |  |  |  |  |  |  |
|  | Vcp | 0.103 |  | Vcp | 0.075 |  | Hprt1 | 0.23 |  | Vcp | 0.46 |  | **Vcp** | 1.19 |
|  | Rplp0 | 0.103 |  | Rplp0 | 0.097 |  | Vcp | 0.25 |  | Rplp0 | 0.47 |  | **Rplp0** | 2.11 |
|  | Ywhaz | 0.175 |  | Ywhaz | 0.162 |  | B2m | 0.28 |  | Ywhaz | 0.48 |  | **Ywhaz** | 3.71 |
|  | B2m | 0.21 |  | B2m | 0.194 |  | Tbp | 0.28 |  | Gusb | 0.49 |  | **B2m** | 4.23 |
|  | Tbp | 0.225 |  | Gusb | 0.2 |  | Rplp0 | 0.31 |  | B2m | 0.49 |  | **Hprt1** | 5.2 |
|  | Gusb | 0.243 |  | Ppia | 0.218 |  | Ppia | 0.36 |  | Ppia | 0.51 |  | Tbp | 5.57 |
|  | Actb | 0.255 |  | Actb | 0.242 |  | Ywhaz | 0.37 |  | Actb | 0.52 |  | Gusb | 6.03 |
|  | Ppia | 0.277 |  | Tbp | 0.256 |  | Reep5 | 0.39 |  | Tbp | 0.52 |  | Ppia | 6.45 |
|  | Hprt1 | 0.292 |  | Hprt1 | 0.261 |  | Ipo8 | 0.39 |  | Hprt1 | 0.54 |  | Actb | 8.47 |
|  | Rpl5 | 0.306 |  | Tfrc | 0.285 |  | Tfrc | 0.39 |  | Tfrc | 0.55 |  | Tfrc | 10.24 |
|  | Tfrc | 0.326 |  | Rpl5 | 0.32 |  | Gusb | 0.4 |  | Ipo8 | 0.56 |  | Ipo8 | 10.93 |
|  | Ipo8 | 0.341 |  | Ipo8 | 0.321 |  | Sdha | 0.43 |  | Rpl5 | 0.56 |  | Rpl5 | 11.45 |
|  | Polr2a | 0.38 |  | Polr2a | 0.576 |  | Ubc | 0.44 |  | Polr2a | 0.71 |  | Sdha | 13.71 |
|  | Sdha | 0.419 |  | Gapdh | 0.604 |  | Actb | 0.44 |  | Sdha | 0.75 |  | Polr2a | 13.9 |
|  | Ubc | 0.45 |  | Sdha | 0.62 |  | Rpl5 | 0.44 |  | Gapdh | 0.75 |  | Reep5 | 14.07 |
|  | Reep5 | 0.477 |  | Ubc | 0.643 |  | Hmbs | 0.53 |  | Ubc | 0.77 |  | Ubc | 15.23 |
|  | Gapdh | 0.507 |  | Psmb4 | 0.655 |  | Polr2a | 0.55 |  | Reep5 | 0.79 |  | Gapdh | 15.92 |
|  | Psmb4 | 0.535 |  | Reep5 | 0.656 |  | Psmb4 | 0.65 |  | Psmb4 | 0.8 |  | Psmb4 | 17.99 |
|  | Hmbs | 0.561 |  | Hmbs | 0.67 |  | Gapdh | 0.65 |  | Hmbs | 0.82 |  | Hmbs | 18.2 |
|  | 18S | 0.586 |  | 18S | 0.733 |  | 18S | 0.73 |  | 18S | 0.85 |  | 18S | 20 |
|  | Pgk1 | 0.65 |  | Pgk1 | 1.197 |  | Pgk1 | 0.88 |  | Pgk1 | 1.26 |  | Pgk1 | 21 |
| **E 14~20/ M1~9 (n=9/18)** | |  |  |  |  |  |  |  |  |  |  |  |  |  |
|  | Rplp0 | 0.236 |  | Rplp0 | 0.068 |  | Reep5 | 0.35 |  | Rplp0 | 0.64 |  | **Rplp0** | **1.78** |
|  | Gapdh | 0.236 |  | Tbp | 0.07 |  | Ubc | 0.35 |  | Tbp | 0.65 |  | **Gapdh** | **3.22** |
|  | Rpl5 | 0.268 |  | Gapdh | 0.248 |  | B2m | 0.39 |  | Gapdh | 0.69 |  | **Tbp** | **3.44** |
|  | Hprt1 | 0.278 |  | Vcp | 0.276 |  | Vcp | 0.43 |  | Gusb | 0.69 |  | **Vcp** | **5.26** |
|  | Tbp | 0.295 |  | Gusb | 0.281 |  | Sdha | 0.49 |  | Rpl5 | 0.71 |  | **Gusb** | **6.03** |
|  | Gusb | 0.319 |  | Rpl5 | 0.371 |  | Ipo8 | 0.51 |  | Vcp | 0.71 |  | Rpl5 | 6.06 |
|  | Ywhaz | 0.35 |  | Hprt1 | 0.409 |  | Tbp | 0.64 |  | Hprt1 | 0.73 |  | Hprt1 | 7.24 |
|  | Vcp | 0.393 |  | Ipo8 | 0.438 |  | Hmbs | 0.69 |  | Ipo8 | 0.78 |  | Ipo8 | 8.06 |
|  | Hmbs | 0.433 |  | Hmbs | 0.455 |  | Polr2a | 0.71 |  | Ywhaz | 0.79 |  | Reep5 | 8.44 |
|  | Psmb4 | 0.47 |  | Polr2a | 0.514 |  | Rplp0 | 0.76 |  | Hmbs | 0.8 |  | Hmbs | 8.97 |
|  | Ipo8 | 0.506 |  | Psmb4 | 0.531 |  | Gusb | 0.81 |  | Polr2a | 0.83 |  | Ubc | 9.46 |
|  | Polr2a | 0.533 |  | Ywhaz | 0.545 |  | Gapdh | 0.84 |  | Psmb4 | 0.84 |  | Polr2a | 10.44 |
|  | Actb | 0.572 |  | Reep5 | 0.669 |  | Psmb4 | 0.86 |  | Reep5 | 0.91 |  | Ywhaz | 10.65 |
|  | Ppia | 0.609 |  | 18S | 0.77 |  | Hprt1 | 0.95 |  | Actb | 0.98 |  | Psmb4 | 11.45 |
|  | Reep5 | 0.649 |  | Actb | 0.835 |  | Rpl5 | 1 |  | 18S | 1.01 |  | B2m | 11.65 |
|  | 18S | 0.683 |  | Ppia | 0.936 |  | 18S | 1.05 |  | Ppia | 1.06 |  | Sdha | 14.67 |
|  | Pgk1 | 0.723 |  | Tfrc | 1.008 |  | Ywhaz | 1.07 |  | B2m | 1.15 |  | Actb | 15.09 |
|  | Tfrc | 0.762 |  | Pgk1 | 1.014 |  | Pgk1 | 1.21 |  | Pgk1 | 1.16 |  | 18S | 15.23 |
|  | B2m | 0.812 |  | B2m | 1.03 |  | Actb | 1.29 |  | Tfrc | 1.18 |  | Ppia | 16.36 |
|  | Ubc | 0.855 |  | Ubc | 1.101 |  | Ppia | 1.42 |  | Ubc | 1.2 |  | Pgk1 | 17.74 |
|  | Sdha | 0.893 |  | Sdha | 1.155 |  | Tfrc | 1.51 |  | Sdha | 1.26 |  | Tfrc | 18.69 |
| **D 1~7/ M1~9 (n=11/18)** | |  |  |  |  |  |  |  |  |  |  |  |  |  |
|  | Reep5 | 0.174 |  | Rplp0 | 0.075 |  | Ubc | 0.18 |  | Rplp0 | 0.48 |  | **Reep5** | 2.38 |
|  | Polr2a | 0.174 |  | Reep5 | 0.085 |  | B2m | 0.32 |  | Reep5 | 0.48 |  | **Rplp0** | 2.58 |
|  | Pgk1 | 0.195 |  | Polr2a | 0.106 |  | Sdha | 0.32 |  | Polr2a | 0.48 |  | **Polr2a** | 2.82 |
|  | Rplp0 | 0.211 |  | Rpl5 | 0.126 |  | Ipo8 | 0.33 |  | Rpl5 | 0.5 |  | **Pgk1** | 5.23 |
|  | Rpl5 | 0.223 |  | Pgk1 | 0.165 |  | Vcp | 0.34 |  | Pgk1 | 0.51 |  | **Rpl5** | 5.89 |
|  | Tbp | 0.248 |  | Tbp | 0.212 |  | Gapdh | 0.5 |  | Tbp | 0.52 |  | Vcp | 6.88 |
|  | Gusb | 0.263 |  | Gusb | 0.249 |  | Polr2a | 0.51 |  | Vcp | 0.53 |  | Tbp | 7.14 |
|  | Vcp | 0.284 |  | Vcp | 0.283 |  | Reep5 | 0.52 |  | Gusb | 0.54 |  | Ipo8 | 7.35 |
|  | Ipo8 | 0.299 |  | Ipo8 | 0.32 |  | Hmbs | 0.52 |  | Ipo8 | 0.55 |  | Ubc | 8 |
|  | Hprt1 | 0.321 |  | Hprt1 | 0.33 |  | Pgk1 | 0.61 |  | Hprt1 | 0.58 |  | Gusb | 8.9 |
|  | Hmbs | 0.345 |  | Hmbs | 0.395 |  | Rplp0 | 0.62 |  | Hmbs | 0.62 |  | Sdha | 9.65 |
|  | Psmb4 | 0.37 |  | Psmb4 | 0.41 |  | Tbp | 0.64 |  | Gapdh | 0.64 |  | Hmbs | 10.46 |
|  | Gapdh | 0.39 |  | Gapdh | 0.455 |  | 18S | 0.66 |  | Psmb4 | 0.64 |  | Gapdh | 10.5 |
|  | 18S | 0.422 |  | Ywhaz | 0.55 |  | Psmb4 | 0.67 |  | Ywhaz | 0.7 |  | Hprt1 | 11.42 |
|  | Sdha | 0.452 |  | 18S | 0.551 |  | Rpl5 | 0.71 |  | 18S | 0.73 |  | B2m | 11.82 |
|  | Ubc | 0.475 |  | Ubc | 0.643 |  | Gusb | 0.73 |  | Ubc | 0.76 |  | Psmb4 | 12.72 |
|  | Ywhaz | 0.5 |  | Sdha | 0.665 |  | Hprt1 | 0.84 |  | Sdha | 0.77 |  | 18S | 14.23 |
|  | B2m | 0.536 |  | Ppia | 0.755 |  | Ywhaz | 0.98 |  | Ppia | 0.85 |  | Ywhaz | 15.65 |
|  | Ppia | 0.572 |  | B2m | 0.888 |  | Ppia | 1.18 |  | B2m | 0.95 |  | Ppia | 18.49 |
|  | Actb | 0.615 |  | Actb | 0.929 |  | Actb | 1.32 |  | Actb | 0.99 |  | Actb | 20 |
|  | Tfrc | 0.664 |  | Tfrc | 1.051 |  | Tfrc | 1.47 |  | Tfrc | 1.14 |  | Tfrc | 21 |
| **D 1~3/ D 4~7 (n=6/5)** | |  |  |  |  |  |  |  |  |  |  |  |  |  |
|  | Vcp | 0.056 |  | Gusb | 0.072 |  | Ipo8 | 0.13 |  | Vcp | 0.23 |  | **Vcp** | 1.78 |
|  | Rplp0 | 0.056 |  | Vcp | 0.08 |  | B2m | 0.14 |  | Gusb | 0.23 |  | **Rplp0** | 2.45 |
|  | Gusb | 0.074 |  | Rpl5 | 0.088 |  | Rplp0 | 0.14 |  | Rplp0 | 0.23 |  | **Gusb** | 2.45 |
|  | B2m | 0.095 |  | Rplp0 | 0.09 |  | Tbp | 0.14 |  | Rpl5 | 0.24 |  | **Ipo8** | 4.6 |
|  | Polr2a | 0.112 |  | Ppia | 0.094 |  | Gusb | 0.15 |  | Ppia | 0.24 |  | **Rpl5** | 4.9 |
|  | Rpl5 | 0.125 |  | Sdha | 0.099 |  | Vcp | 0.15 |  | Sdha | 0.24 |  | B2m | 6.45 |
|  | Sdha | 0.133 |  | Ipo8 | 0.102 |  | Hprt1 | 0.17 |  | Hprt1 | 0.25 |  | Sdha | 7.09 |
|  | Ipo8 | 0.141 |  | Hprt1 | 0.107 |  | Polr2a | 0.17 |  | Ipo8 | 0.25 |  | Ppia | 7.24 |
|  | Hprt1 | 0.149 |  | Actb | 0.124 |  | Sdha | 0.17 |  | B2m | 0.25 |  | Hprt1 | 7.71 |
|  | Ppia | 0.153 |  | Reep5 | 0.125 |  | Rpl5 | 0.17 |  | Polr2a | 0.26 |  | Polr2a | 8.39 |
|  | Reep5 | 0.157 |  | Polr2a | 0.128 |  | Ppia | 0.18 |  | Reep5 | 0.26 |  | Tbp | 9.06 |
|  | Actb | 0.161 |  | B2m | 0.133 |  | Ywhaz | 0.18 |  | Actb | 0.26 |  | Reep5 | 11.2 |
|  | Ywhaz | 0.169 |  | Ywhaz | 0.206 |  | Reep5 | 0.19 |  | Ywhaz | 0.29 |  | Actb | 11.81 |
|  | Pgk1 | 0.182 |  | Pgk1 | 0.217 |  | Ubc | 0.19 |  | Pgk1 | 0.31 |  | Ywhaz | 12.74 |
|  | Tbp | 0.196 |  | Tbp | 0.269 |  | Actb | 0.21 |  | Tbp | 0.34 |  | Pgk1 | 14.48 |
|  | Gapdh | 0.21 |  | Gapdh | 0.289 |  | Pgk1 | 0.26 |  | Gapdh | 0.36 |  | Ubc | 16.66 |
|  | Ubc | 0.225 |  | 18S | 0.313 |  | 18S | 0.26 |  | 18S | 0.38 |  | Gapdh | 16.7 |
|  | 18S | 0.237 |  | Ubc | 0.325 |  | Tfrc | 0.33 |  | Ubc | 0.38 |  | 18S | 17.24 |
|  | Tfrc | 0.253 |  | Tfrc | 0.345 |  | Gapdh | 0.35 |  | Tfrc | 0.41 |  | Tfrc | 18.74 |
|  | Psmb4 | 0.283 |  | Psmb4 | 0.529 |  | Hmbs | 0.36 |  | Psmb4 | 0.57 |  | Psmb4 | 20.25 |
|  | Hmbs | 0.314 |  | Hmbs | 0.575 |  | Psmb4 | 0.4 |  | Hmbs | 0.61 |  | Hmbs | 20.75 |
| **M1~9 (n=18)** | |  |  |  |  |  |  |  |  |  |  |  |  |  |
|  | Polr2a | 0.102 |  | Reep5 | 0.039 |  | Ubc | 0.17 |  | Reep5 | 0.28 |  | **Reep5** | 2.45 |
|  | Sdha | 0.102 |  | Sdha | 0.047 |  | Vcp | 0.24 |  | Sdha | 0.28 |  | **Sdha** | 2.69 |
|  | Reep5 | 0.118 |  | Ppia | 0.073 |  | B2m | 0.25 |  | Ppia | 0.29 |  | **Polr2a** | 3.76 |
|  | Ywhaz | 0.13 |  | Rplp0 | 0.079 |  | Ipo8 | 0.31 |  | Rplp0 | 0.29 |  | **Ppia** | 5.3 |
|  | Gusb | 0.137 |  | Polr2a | 0.108 |  | Gusb | 0.33 |  | Polr2a | 0.29 |  | **Rplp0** | 6.16 |
|  | Ipo8 | 0.14 |  | Ywhaz | 0.115 |  | Tbp | 0.33 |  | Tbp | 0.29 |  | Ywhaz | 6.24 |
|  | Tbp | 0.143 |  | Hmbs | 0.119 |  | Hmbs | 0.35 |  | Ywhaz | 0.3 |  | Gusb | 6.51 |
|  | Ppia | 0.149 |  | Tbp | 0.123 |  | Polr2a | 0.36 |  | Gusb | 0.3 |  | Tbp | 6.7 |
|  | Rplp0 | 0.152 |  | Gusb | 0.137 |  | Ywhaz | 0.37 |  | Hmbs | 0.3 |  | Ipo8 | 7.33 |
|  | Hmbs | 0.155 |  | Hprt1 | 0.144 |  | Rplp0 | 0.38 |  | Ipo8 | 0.31 |  | Hmbs | 8.15 |
|  | Hprt1 | 0.164 |  | Pgk1 | 0.152 |  | Reep5 | 0.39 |  | Hprt1 | 0.32 |  | Vcp | 8.9 |
|  | Pgk1 | 0.174 |  | Ipo8 | 0.164 |  | Ppia | 0.39 |  | Pgk1 | 0.34 |  | Ubc | 9.1 |
|  | Rpl5 | 0.183 |  | Rpl5 | 0.166 |  | Sdha | 0.41 |  | Rpl5 | 0.34 |  | Hprt1 | 11.41 |
|  | Vcp | 0.192 |  | Actb | 0.236 |  | Hprt1 | 0.43 |  | Vcp | 0.35 |  | B2m | 11.5 |
|  | Actb | 0.203 |  | Gapdh | 0.242 |  | Actb | 0.43 |  | Actb | 0.38 |  | Pgk1 | 12.81 |
|  | Gapdh | 0.215 |  | Vcp | 0.245 |  | Rpl5 | 0.48 |  | Gapdh | 0.39 |  | Rpl5 | 13.69 |
|  | Psmb4 | 0.237 |  | Psmb4 | 0.356 |  | Pgk1 | 0.49 |  | Psmb4 | 0.47 |  | Actb | 14.74 |
|  | B2m | 0.261 |  | B2m | 0.471 |  | Psmb4 | 0.52 |  | B2m | 0.52 |  | Gapdh | 16.44 |
|  | Ubc | 0.281 |  | Ubc | 0.473 |  | Gapdh | 0.54 |  | Ubc | 0.53 |  | Psmb4 | 17.24 |
|  | 18S | 0.321 |  | 18S | 0.625 |  | 18S | 0.72 |  | 18S | 0.7 |  | 18S | 20 |
|  | Tfrc | 0.402 |  | Tfrc | 1.15 |  | Tfrc | 1.25 |  | Tfrc | 1.17 |  | Tfrc | 21 |
|  |  |  |  |  |  |  |  |  |  |  |  |  |  |  |
