# Supplementary figures and images for: Identification of reference genes for gene expression studies among different developmental stages of murine hearts
Source: BMC Dev Biol. 2021 Sep 8;21:13. doi: 10.1186/s12861-021-00244-6 (PMC8425138; doi:10.1186/s12861-021-00244-6)

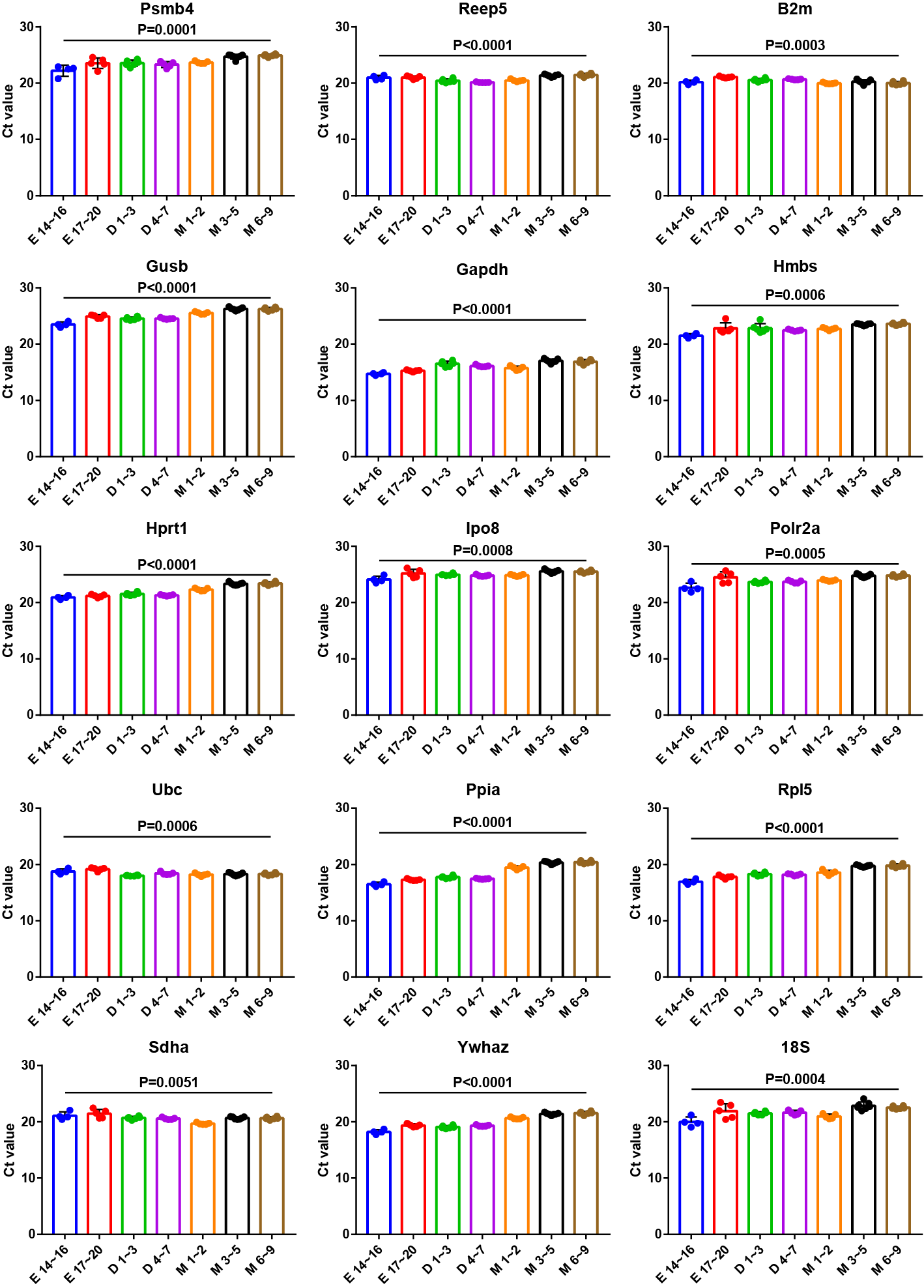

Supplement: Supplementary file 3 — Additional file 3: Figure S1. The expression variabilities of 15 housekeeping genes at 7 different developmental stages were plotted with bar and scatter graphs. Values are given as raw cycle threshold (Ct) values, mean and standard deviation of the Ct values were indicated in the plot. [file 12861_2021_244_MOESM3_ESM.tif]
